# Vaccination Officers' Grievances

**Published:** 1911-08-12

**Authors:** 


### Vaccination Officers' Grievances.

The question of the payment of vaccination officers, which was recently brought up in the House of Commons, exposes a very real grievance. These officers are paid a certain sum for every vaccination within their districts, and their duty is to see that the operation is duly carried out. In poor districts the work at the best is tiresome, and takes an abundance of both time and good temper. But of late years the position of the vaccination officer has become almost intolerable. The people he has to deal with have been practically taught to regard him as an enemy, or at least as the enemy's emissary. When, as the law compels, he has to prosecute parents who have neither had their children vaccinated nor taken the necessary steps to obtain exemption, he has been treated as if he were a determined enemy of the poor. At present the vaccination officer is not quite so much out of favour with those with whom he has to deal, because they have become expert in the exemption business and prosecutions are rarer. But it is still his business to see that children are either vaccinated or formally exempted, and his salary is still dependent on the fees for vaccinations. This means that his income has diminished while his duties have not, and they may even have increased. Not only present income but future prospects are affected by the social tendencies of the day. For the pension of a vaccination officer is calculated on a five years' average of his receipts, and therefore, though a man may have been doing his duty faithfully in a populous neighbourhood, if the neighbourhood happens to be an anti-vaccination one, where his work has been hard and thankless, he will receive less than in another where it has been easy and successful. Mr. Burns' statement that it would not be practicable to pay the vaccination officers by a fixed salary instead of fees, without largely increasing the size of the districts to be dealt with by one officer, is true enough; but if vaccination officers are to continue—which, in an age when exemption seems so easy that it may soon become universal, is an open question—it is necessary to provide a secure payment of reasonable amount for them if their services are required at all.

